# Positional cloning identified *HvTUBULIN8* as the candidate gene for round lateral spikelet (RLS) in barley (*Hordeum vulgare* L.)

**DOI:** 10.1007/s00122-023-04272-7

**Published:** 2023-01-19

**Authors:** Dandan Qin, Gang Liu, Rui Liu, Chunchao Wang, Fuchao Xu, Qing Xu, Yu Ling, Guoqing Dong, Yanchun Peng, Shuangtao Ge, Ganggang Guo, Jing Dong, Chengdao Li

**Affiliations:** 1 Institute of Food Crops, Hubei Academy of Agricultural Sciences, Wuhan, 430064 Hubei China; 2 Key Laboratory for Crop Molecular, Breeding of Ministry of Agriculture and Rural Affairs, Wuhan, 430064 Hubei China; 3 Wuhan Polytechnic University, Wuhan, 430023 Hubei China; 4 Institute of Crop Science, Chinese Academy of Agricultural Sciences, Beijing, 100081 China; 5 College of Coastal Agricultural Sciences, Guangdong Ocean University, Zhanjiang, 524088 Guangdong China; 6 Western Crop Genetics Alliance, College of Science, Health, Engineering and Education, Murdoch University, Western Australia, WA 6150 Australia

## Abstract

**Key message:**

Map-based cloning, subcellular localization, virus-induced-gene-silencing and transcriptomic analysis reveal *HvTUB8* as a candidate gene with pleiotropic effects on barley spike and leaf development via ethylene and chlorophyll metabolism.

**Abstract:**

Barley lateral spikelet morphology and grain shape play key roles in grain physical quality and yield. Several genes and QTLs for these traits have been cloned or fine mapped previously. Here, we report the phenotypic and genotypic analysis of a barley mutant with *round lateral spikelet (rls)* from *cv.* Edamai 934. *rls* had round lateral spikelet, short but round grain, shortened awn, thick glume and dark green leaves. Histocytologic and ultrastructural analysis revealed that the difference of grain shape of *rls* was caused by change of cell arrangement in glume, and the dark leaf color resulted from enlarged chloroplast. *HvTUBULIN8* (*HvTUB8*) was identified as the candidate gene for *rls* by combination of RNA-Seq, map-based-cloning, virus-induced-gene-silencing (VIGS) and protein subcellular location. A single G-A substitution at the third exon of *HvTUB8* resulted in change of Cysteine 354 to tyrosine. Furthermore, the mutant isoform Hvtub8 could be detected in both nucleus and cytoplasm, whereas the wild-type protein was only in cytoplasm and granular organelles of wheat protoplasts. Being consistent with the rare phenotype, the “A” allele of *HvTUB8* was only detected in *rls*, but not in a worldwide barley germplasm panel with 400 accessions. VIGS confirmed that *HvTUB8* was essential to maintain spike integrity. RNA-Seq results suggested that *HvTUB8* may control spike morphogenesis via ethylene homeostasis and signaling, and control leaf color through chlorophyll metabolism. Collectively, our results support *HvTUB8* as a candidate gene for barley spike and leaf morphology and provide insight of a novel mechanism of it in barley development.

**Supplementary Information:**

The online version contains supplementary material available at 10.1007/s00122-023-04272-7.

## Introduction

Barley (*Hordeum vulgare* L.) is the fourth largest cereal crop worldwide. As a staple food of ancient civilizations, domesticated from *Hordeum vulgare subsp. Spontaneum* ~ 10,000 years ago (Harlan et al. 1973; Zohary and Hopf 2000), cultivated barley is now used mainly for animal feed and malting. Selection of varieties with high yield potential is always the ultimate goal for barley breeding. Inflorescence architecture is one of the key factors that affect grain yield in cereal crops, such as rice, maize, barley and wheat (Wang et al. 2021a). Among them, barley and wheat usually form typical compact and branchless “spike” inflorescences, characterized by sessile spikelets produced directly from alternating, opposing nodes on the branchless main axis or rachis, which is specific to the tribe *Triticeae*, while inflorescences of maize and rice have highly branched tassels or panicles (Koppolu et al. 2013). Moreover, being different from its close relative wheat, barley inflorescence comprises a multi-node central stalk (rachis) with tripartite clusters of unifloretted spikelets attached alternately along its length. This distinctive triple spikelet arrangement arises when each inflorescence node forms a triple spikelet meristem (TSM) that cleaves into a large central spikelet meristem (CSM) bordered by two smaller lateral spikelet meristems (LSMs) (Zwirek et al. 2019). Relative fertility of lateral spikelets within each cluster leads to spikes with two or six row of spike, or an intermediate morphology. Two-rowed spikes bear a single grain from a fertile central spikelet flanked by two sterile lateral spikelets within each rachis node, while all three spikelets are fertile and develop into grains in six-rowed barley (Ramsay et al. 2011a). The two-rowed state is ancestral, being found in the wild progenitor of cultivated barley (*Hordeum vulgare* ssp. *Spontaneum*), where the sterile spikelets form part of the seed dispersal mechanism (Zohary and Hopf 2000).

Up to date, five major genes, namely *SIX-ROWED SPIKE* (*VRS*) genes, controlling lateral spikelet (LS) development has been identified by forward genetics, including *VRS1*, *VRS2*, *VRS3* (syn. *INTERMEDIUM-A*), *VRS4* (syn. *INTERMEDIUM-E*) and *VRS5* (syn. *INTERMEDIUM-C*). *VRS1* on chromosome 2H encodes a homeodomain-leucine zipper class I protein (HD-ZIP1) (He et al. 2004; Komatsuda et al. 2007). *VRS2* mutant can develop supernumerary spikelets at its base and occasionally enlarged and fertile LSs toward the center of the spike, but almost two-rowed-like, sterile LSs at its tip. *VRS2* is on the long arm of chromosome 5H, encoding a homologue of the *Arabidopsis* SHORT INTERNODES (SHI) transcriptional regulator (Youssef et al. 2017). Barley two-rowed (in the lower portion of the spike) and six-rowed (in the upper part) spikes-controlled gene *VRS3* encodes a putative Jumonji C-type H3K9me2/me3 demethylase, which is on the short arm of chromosome 1H (Bull et al. 2017). Apart from lateral spikelet fertility, *vrs4* mutants show indeterminate triple spikelet meristems, thereby producing additional spikelets/florets. *VRS4* is on the short arm of chromosome 3H and encodes a LATERAL ORGAN BOUNDARIES (LOB) transcription factor orthologous to the maize *RAMOSA2* gene, which prevents ectopic branching in maize ears and tassels (Koppolu et al. 2013). *VRS5* on short arm of chromosome 4H encodes a class II TCP transcription factor whose homologues in maize, rice, wheat and *Arabidopsis* repress tiller bud outgrowth to promote apical dominance (Ramsay et al. 2011b). The re-sequence analysis of *HvMADS-box* family genes in 30 spike-contrasting barley lines revealed that *HvMADS56* also played an important role in lateral spikelet development (Sayed et al. 2021).

Grain shape is another essential factor for barley grain physical quality and yield. In wheat, a single amino acid substitution in STKc_GSK3 kinase conferred semispherical grains of *Triticum sphaerococcum* (Cheng et al. 2020). A number of QTLs for barley grain shape determinants (grain length, grain width, grain thickness) had been mapped throughout the whole genome (Fang et al. 2020; Wang et al. 2019a, 2021b). Some major QTLs have been fine mapped, such as *qGL5H* (Watt et al. 2019) and *qGL2H* (Watt et al. 2020) for grain length locus. However, the genetic and biological basis of round grain development still remains largely unknown.

Typically, barley has long awns, but short-awn variants exist. Awn plays an important role in seed dispersal, protection as well as photosynthesis and yield (Abebe et al. 2009; Elbaum et al. 2007), whereas, awnless and short awn cultivars are likely to perform better under extreme conditions such as drought (Rebetzke et al. 2016). Given the potential impact of awn length on barley yield, a number of QTLs affect this trait have been identified on barley genome, both from modern cultivars (Chen et al. 2012; Gyenis et al. 2007) and wild accessions (Liller et al. 2017). Three awnless-specific genes have so far been cloned in barley. One was *Knox3* transcription factor for the hood-shaped awn (Müller et al. 1995), and mutation of the *SHI*-family transcription factor *lks2* produced awns that were only a half of the normal ones (Yuo et al. 2012), while *vrs1* was allelic to the *reduced lateral spikelet appendage* (*lr*) locus, characteristic of having normal and long awns on the central spikelets and awnless on the lateral spikelets (Komatsuda et al. 2007). Genetic analysis further revealed that the loci/genes underling barley awn trait diversity could interact with each other (Huang et al. 2020).

Previously, a mutant library of Edamai 934 (referred to E934 in this study) was constructed by combined ^60^Co-γ and EMS treatment. E934 is a two-rowed feeding barley variety bred by Hubei Academy of Agricultural Sciences (Hubei, China) (Qin et al. 2021). The barley mutant *Round Lateral Spikelets* (*rls*), which was identified from the E934 mutant library, exhibits dark leaf color, round lateral spikelet, round grain, shortened awn and abnormal glume. Here, we identified the causal gene through positional cloning. A G-A nucleotide substitution in *HvTUB8* gene may contribute to the pleiotropic changes on barley spike and leaf morphology in *rls*. The current study will not only lay a foundation for unraveling the function and mechanisms of *HvTUB8* in spike and leaf development, but also be useful for barley breeding using *rls* and *HvTUB8* as a resource.

## Materials and methods

### Plant materials

**Fig. 1:**
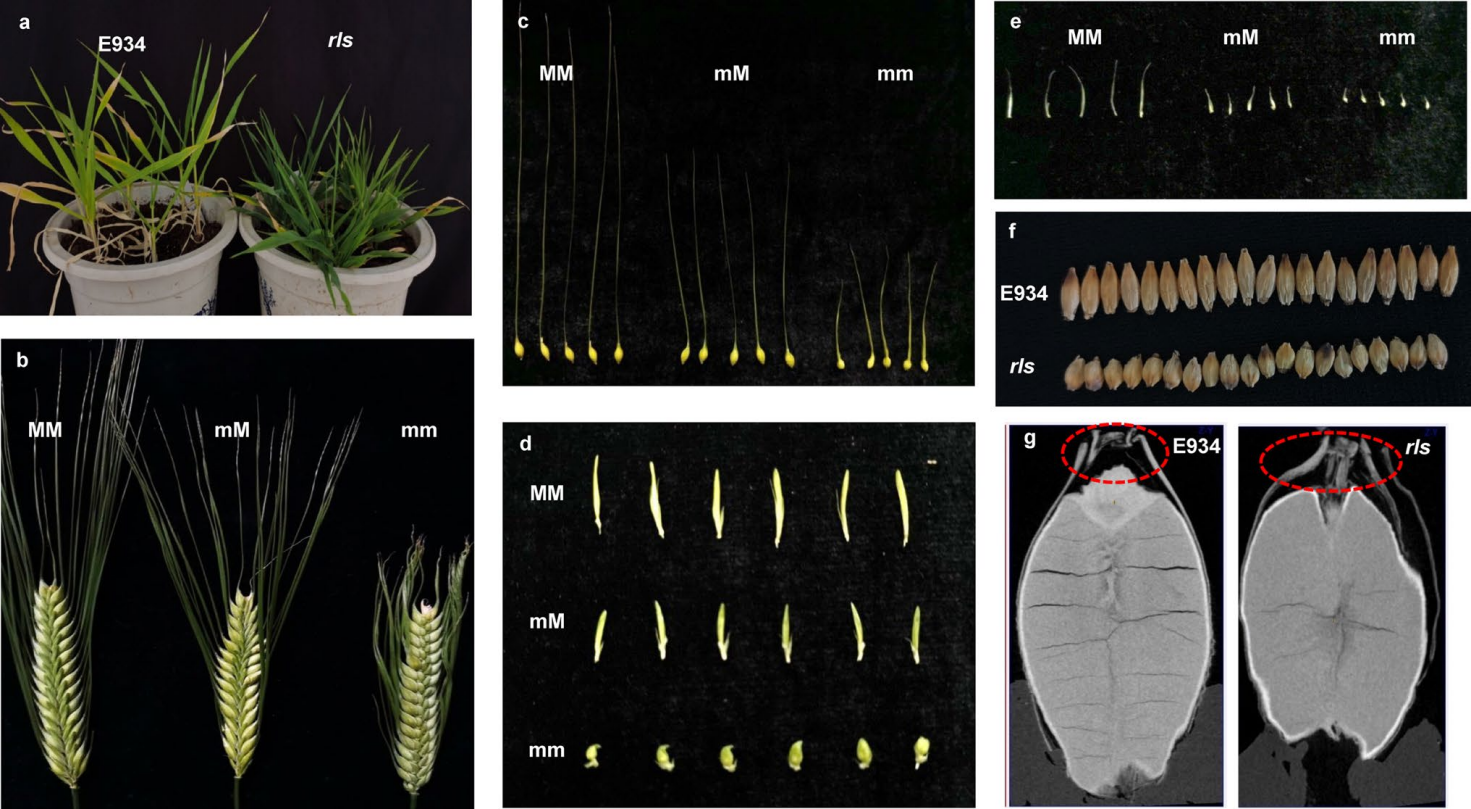
Seedling and spike morphology of *rls*. **a** Seedling of E934 and *rls*; **b** Wild-type (MM), medium mutant (mM) type and mutant (mm) type spikes; **c** central spikelets; **d** lateral spikelet; **e** bilateral empty glumes; **f**, **g**: seeds of E934 and *rls*

*Rls* mutant was identified from ^60^Co-γ and EMS induced E934 mutant library in our previous work, which was mainly characterized as a mutant with abnormal spike morphology. *rls* was crossed with Yan 03174, a typical two-rowed barley variety, which was bred by Yancheng Academy of Agricultural Sciences (Jiangsu, China). Genetic characterization of the gene (s) controlling the *rls* phenotype was analyzed based on the spike morphology of F_1_ hybrid, F_2_ population and F_2:3_ families. Chi-squared test was used to determine the suitability of observed data with expected segregation ratios. All of the field work was conducted in the experimental station of Hubei Academy of Agricultural Sciences (Wuhan, China). Relative chlorophyll concentration in the fully developed leaf on the main tiller of *rls* and E934 was measured using SPAD-502 at the booting stage (Fig. 1a). Average of five leaves for *rls* and E934 were measured and compared. Dried seeds of *rls* and E934 were subjected to micro-CT scanning (SkyScan 1275, Bruker) to evaluate differences in three dimensions.

### Scanning electron microscopy

Immature spike (3 mm to 5 mm) and seedling leaves from *rls* and E934 were pre-fixed with 2.5% glutaraldehyde in phosphate buffer (PH = 7.0) at 4 ℃ overnight and further treated as described in our previous work (Qin et al. 2015). The specimens were observed under a Hitachi HT7700 scanning electron microscope (Hitachi, Japan).

### Plant softening paraffin embedding experiment

Intact glumes of E934 and *rls* were picked from the middle of the spike when it extracted from flag leaf completely. Then glumes were fixed in FAA at 4 ℃ overnight. Subsequent softening, dehydration, paraffin embedding, splicing and statistical analysis were conducted by Servicebio® (Wuhan, China) according to the standard procedure.

### BSA based on RNA-Seq (BSR)

Young leaves of 60 mutant type individuals and 60 wild-type individuals were picked up from 60 heterozygous F_2:3_ families and pooled together to construct the mutant pool and wild-type pool, respectively. RNA was extracted using TRIzol (Rio et al. 2010). RNA-Seq was carried out on an Illumina HiSeq 2500 platform following the manufacturer’s protocol. Both RNA extraction and RNA-Seq were conducted by Beijing Novogene Bioinformatics Technology Co. Ltd. Raw RNA-Seq reads were assessed for quality control by Trimmomatic v 0.32 (Bolger et al. 2014) after removing adapter sequences and low-quality bases. Adapter sequences were removed by identifying the pairing part between the two paired-end reads, followed by trimming bases that were not paired at read ends. Low-quality bases at read ends whose quality values (Phred-value) were lower than 3 were trimmed, and low-quality bases in the middle of each read were identified by a 10 bps sliding window method (mean Phred-value < 15 in the window) (Wang et al. 2017a, b). High-quality reads were aligned to the barley *cv.* Morex reference genomic sequence (2016 version 1) (Mascher et al. 2017). After alignments, the mapping results were filtered using an in-house Perl script. Only uniquely mapped reads with Phred-value quality larger than 40 were kept. Variant calling and subsequent identification of variants possibly linked to the target gene was carried using the Haplotype Caller module in GATK v3.2–2 as previously described in wheat (Wang et al. 2017a, b; Zhang et al. 2020). Briefly, the read count information for each allele per variant was obtained from the GATK output. Then, a Fish exact test was applied on read counts for each variant site (Indels and SNPs) between the two bulks to identify variants possibly linked to the target gene. Additionally, the allele frequency for each variant and the allele frequency difference (AFD) between the two bulks were calculated. Variants with *p* < 1e−10 and AFD > 0.6 were considered to be putatively target gene linked (Wang et al. 2017a, b; Hua et al. 2020).

### DNA isolation

Genomic DNA of E934, *rls* and Yan 03174 was extracted from young leaves using TaKaRa MiniBEST Universal Genomic DNA Extraction Kit following the manufacture’s instruction. Genomic DNA of the F_2_, F_3_ and F_4_ plants derived from Yan 03174 and *rls* was extracted from young leaves using CTAB method.

### Genotyping based on Penta-primer amplification refractory mutation system (PARMS)

Indel and SNP markers were developed according to the BSR analysis. These markers were firstly used to screen Yan 03174 and *rls*. Then polymorphic markers were used to genotype the F_2_, F_3_ and F_4_ plants derived from Yan 03174 and *rls* based on PARMS (Lu et al 2020). All PARMS analysis was conducted by Gentides Biotech Co., Ltd (Wuhan, China). Five μl PCR system contained 2.5 μl 2 × PARMS PCR mix, 150 nM each allele-specific primer, 400 nM locus-specific primer, and 1.4 μl alkaline lysis DNA template. The thermal cycler program included denaturing at 95 °C for 15 min and then 10 cycles of denaturation at 95 °C for 20 s and annealing started at 65 °C for 1 min and then decreasing 0.8 °C per cycle to the annealing temperature at 57 °C. This was followed by 32 cycles of denaturation at 95 °C for 20 s and annealing at 57 °C for 1 min. The well plate was read using a TECAN infinite M1000 plate reader; SNP calling and plots were carried out by an online software snpdecoder (http://www.snpway.com/snpdecoder/) combining manual modification. Linkage analysis was conducted using Mapmaker 3.0 and a LOD score threshold of 3.0 (Lander et al. 1987).

### Analysis of linked markers and cloning of candidate genes

Sequences of flanking markers were conducted by BLASTN analysis online (https://galaxy-web.ipk-gatersleben.de/) (MorexV3 assembly) (Mascher et al. 2021) to get the updated genomic region containing the gene. High-confident genes in the target region were extracted according to MorexV3 assembly. Several gene-specific overlapped primers were designed based on the genomic sequence of these genes using DNAMAN5.0 software. Specific PCR products from genomic DNA of E934, *rls* and Yan 03174 were sequenced and compared with each other. cDNA from 5 to 10 mm young panicle of *rls* and E934 was used to clone CDS of *HvTUB8*. 1000bps upstream region of the candidate gene was also sequenced from E934 and *rls* according to the reference genomic sequence of *cv.* Morex. All the PCRs were conducted using TaKaRa Premix TaqTM (Japan). 20 μl PCR included 2 × TaKaRa Premix 10 μl, 10 μM Forward primer 0.4 μl, 10 μM Reverse primer 0.4 μl, DNA template 10–100 ng, PCR water several μl. PCR mixtures were subjected to Touch-down PCR program (95 °C 5 min; ten cycles of 95 °C 30 s, 62–57 °C (− 0.5 °C /cycle) 45 s, 72 °C 1–2 min; 35 cycles of 95 °C 30 s, 57 °C 45 s; 72 °C 1–2 min; 72 °C 5 min) on ABI VeritiPro PCR machine. Specific PCR products with expected size were subjected to Sanger sequencing. Secondary structure of proteins was predicted by DNAMAN5.0.

### Phylogenetic analysis of *HvTUB8* homologue genes

Homologue genes of *HvTUB8* in other gramineae species were downloaded from NCBI according to BLASTP results. Accession numbers of other barley *β-TUBULIN* genes were extracted according to BLASTN search in database “Barley all CDS Morex V3.0 (Jul 2020)” (https://galaxy-web.ipk-gatersleben.de/), and then, sequences were downloaded from “BARLEX” (https://apex.ipk-gatersleben.de/apex/f?p=284:10::::::)*.* Sequences of OsTUB proteins were also extracted from NCBI according to their accession numbers (Yoshikawa et al. 2003). Alignment and phytogenetic tree of these *HvTUB8* homologue genes was constructed based on their protein sequences using DNAMAN5.0.

### Spatio-temporal expression of *HvTUB8* in barley

Quantitative Real-Time PCR was performed to analyze expression of *HvTUB8* in different organs and tissues of E934, including leaf at three-leaf stage (seedling leaf); internode below the spike (internode); young panicle (0.5–1 cm, > 1 cm); glume, stamen, pistil, awn and flag leaf at heading stage; grain at 10 DAP (10DAP). Gene-specific primers for *HvTUB8* are shown in Supplementary Table S1. Barley gene *Actin 1* was used as an endogenous control.

### Genetic variation of *HvTUB8* in barley accessions

Allelic variation of *HvTUB8* was analyzed in the 20 accessions according to pan-genome data (Jayakodi et al. 2020), including 1000 bps upstream and downstream regions as well as coding region of B1K-04-12 (FT11), HOR 21599, HOR7552, HOR 13942, OUN333, HOR 3365 (HHOR), HOR 10350, ZDM02064 (CHIBA), HOR 9043, ZDM01467 (DULIHUANG), HOR 3081, HOR 13821, Morex, HOR 8148, Akashinriki, Igri, Hockett, Barke, RGT Planet and Golden Promise (Jayakodi et al. 2020). Genotyping of the gene-specific maker was also conducted on a barley natural population consisting of 400 landraces and modern cultivars with typical two-rowed or six-rowed spikes from worldwide based on PARMS, including China, Australia, Canada, USA and so on.

### Barley stripe mosaic virus (BSMV) virus-induced gene silencing (VIGS)

BSMV-VIGS was used for functional characterization of *HvTUB8* in E934. Specific primers for *HvTUB8* (Supplementary Table S1) were designed to amplify the target fragments of 296 bps region in the first exon, which was inserted into the digested BSMV-γ genome to generate the recombinant BSMV:*HvTUB8* construct. Infection with a BSMV-*TaPDS* control indicated infection and gene-silencing efficiency by bleaching of leaf color. Following inoculation and quantification were conducted as described before (Shang et al. 2020). Six plants of E934 were infected at two-leaf seedling stage for both BSMV:*HvTUB8* and BSMV control. When bleaching was visible in BSMV-*TaPDS*-infected plants, Real-time PCR was conducted to analyze the expression of the target gene in the newly developed leaves after BSMV:*HvTUB8* infection as mentioned above.

### Subcellular localization analysis

Complete ORF of wild-type *HvTUB8* in E934 and the variant in *rls* (designed as *Hvtub8* in this study) was amplified with gene-specific primers (Supplementary Table S1), and the product was digested by *Eco31I* and cloned into the expression cassette of modified pCAMBIA1302 (Biorun, Wuhan, China) to generate pCAMBIA1302-35S::HvTUB8-eGFP and pCAMBIA1302-35S::Hvtub8-eGFP vectors, respectively. The construction was confirmed by enzyme digestion and sequencing. The confirmed constructs and vector alone were transformed into wheat protoplasts via co-cultured with PEG4000 (Yoo et al. 2007). The GFP signal was observed by Confocal Laser Scanning Fluorescence Microscopy after 18–24-h incubation in dark (LSFM, Carl Zeiss).

### Transcriptome analysis

Immature spikes from *rls* and E934 at glume primordium (GP) to lemma primordium (LP) formation stage (about 0.5–1 cm) were collected and frozen in liquid nitrogen and then stored at − 80 ℃, three biological repeats for both E934 and *rls*. Total RNA extraction, library construction and RNA-seq were performed by Beijing Novogene Bioinformatics Technology Co. Ltd (Beijing, China) as mentioned above. The clean reads of each sample were mapped to MorexV3 assembly (Mascher et al. 2021). Transcript quantification from RNA-Seq data was analyzed using the Salmon (1.9.0) tool. Expression levels of mapped reads were quantified as Transcripts Per Kilobase of exon model per Million mapped reads (TPM) (Patro et al. 2017). The Bioconductor package DESeq2 (Love et al. 2014) was used to perform differential expression analysis. Differential expression genes (DEGs) between *rls* and E934 were defined as |log2 fold change (LFC)|≥ 1.5, adjusted *P* ≤ 0.05. GO Enrichment analysis of DEGs was carried out online (http://wheat.cau.edu.cn/TGT/) (Chen et al. 2020). GO terms with a corrected FDR of < 0.05 were considered to be significantly enriched. The percentage of DEGs for each GO term was calculated with reference to all transcripts in MorexV3 assembly. Expression of the genes in ethylene and chlorophyll pathways was illustrated by indoor perl script.

## Results

### Phenotypic analysis of barley *rls* mutant

The mutant *rls* was initially identified from screening of ^60^Co-γ and EMS-treated mutant library in the genetic background of E934. Leaf color of *rls* was much darker than that of E934 (Fig. 1a) during the whole life, and chlorophyll concentration reflected by SPAD was 29.03 ± 1.11 and 52.53 ± 3.25 in E934 and *rls* at the booting stage, respectively. Besides, *rls* was mainly characterized as a mutant with abnormal spike morphology (Fig. 1b). Lateral spikelets of *rls* were round and sterile. In addition, awn of *rls* was very short, glume was harder, bilateral empty glumes were also much shorter but a little bit wider than wild type (Fig. 1c–e). Grains of *rls* were round and shorter than wild type, but there was no significant difference on grain width between *rls* and E934 (Fig. 1f). Consequently, thousand grain weight of *rls* was only 32.26 g, being much lower than 45.86 g of E934. Moreover, outer glume section around the embryo in dried seeds of *rls* (166.40 μm) was much thicker than that in E934 (108.00 μm) (Fig. 1g). *rls* was also characterized as dwarf, plant height of which was about 60% of wild type and heading date of *rls* was almost 14 days later than E934.

**Fig. 2:**
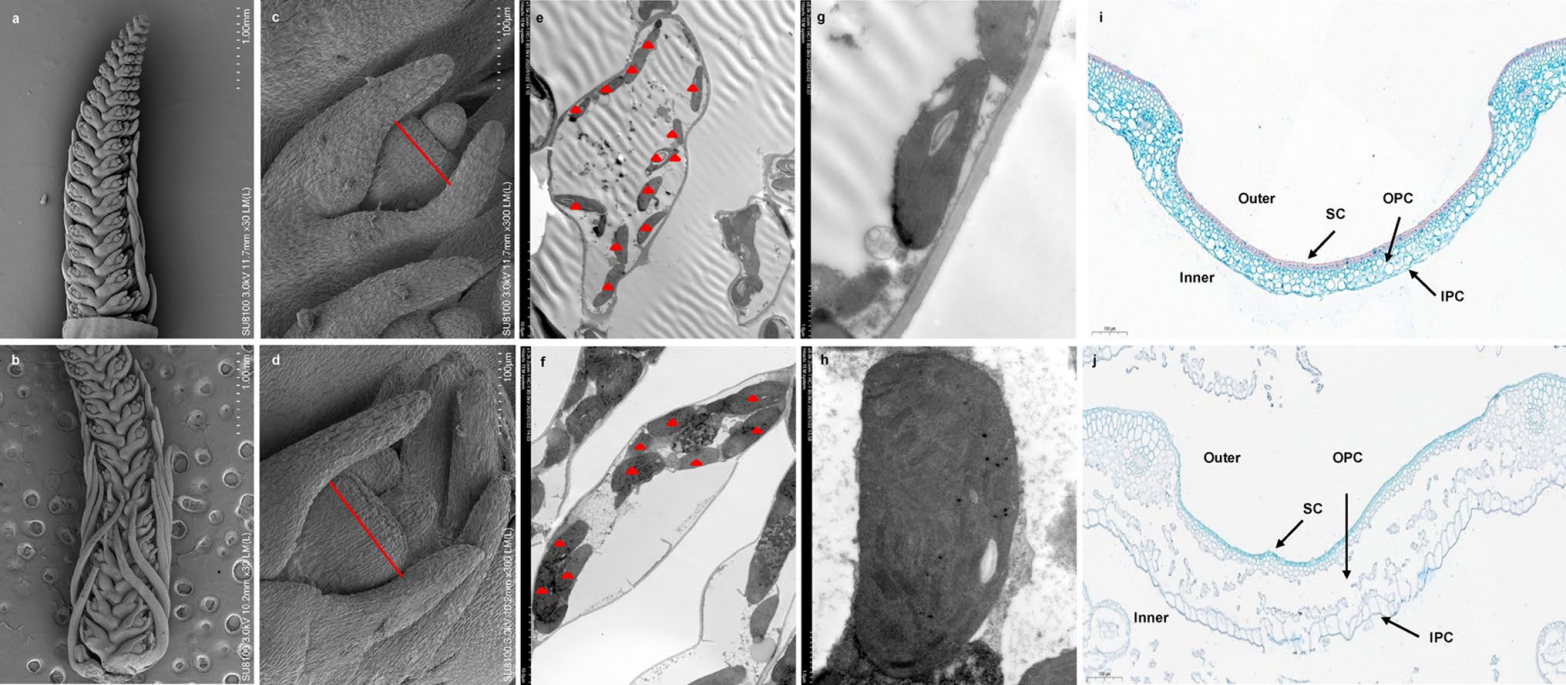
Immature spike and ultrastructure of chloroplast from E934 and *rls*. **a**, **b** 5-mm spike of E934 and *rls*; **c**, **d** lateral spikelet of E934 and *rls*; **e**, **f** ultrastructure of leaf cell from E934 and *rls*; **g**, **h** chloroplast of E934 and *rls*; **i**, **j**: horizontal section of lemma from E934 and *rls* (bar = 100 μm). SC, silicified cells; OPC, outer parenchyma cells; IPC, inner parenchyma cells

To find out which stage was crucial for the differentiation of *rls* spike morphology, 3 mm and 5 mm young panicles of E934 and *rls* were scanned under electron microscope. It was found that there was no difference between 3 mm spikes of E934 and *rls.* However, for 5 mm spikes, *rls* awn was longer than E934, especially the awn at the bottom (Fig. 2a, b), though awn of *rls* was much shorter than E934 at maturity stage. Despite these differences, lateral spikelets clearly increased in size with enlarged lemmas enclosing differentiating floral organs in *rls* as compared with E934 (Fig. 2c, d). What is more, to determine whether dark green leaf color of *rls* was associated with development of chloroplast, ultrastructure of chloroplast of E934 and *rls* leaves at booting stage was also investigated under scanning electron microscope. Consistently, chloroplast size was increased in *rls* as compared with that in E934, almost half of the cell being occupied by chloroplasts in *rls* (Fig. 2e–h).

To further reveal the cellular basis of round grain in *rls* mutant, cross section of hull in E934 and *rls* was observed. Significant differences of cell morphology were observed between E934 and *rls* both longitudinally and horizontally. As shown in Fig. 2, a regular pattern of three cell layers including silicified cells (SC), outer parenchyma cells (OPC) and inner parenchyma cells (IPC) was observed in WT (Fig. 2i, j). There was no significant difference between SC of E934 and *rls*; however, a remarkable difference was observed between OPC and IPC between them. Wider empty region in the OPC layer but a relative bigger and regular IPC was observed both in the lemmas and paleas from *rls* as compared with that from E934, which may contribute to the thicker glumes and round grains in *rls* (Fig. 1g).

### Genetic dissection of *rls* mutant underling gene

To decipher genetic feature of the causal gene, *rls* was crossed with Yan 03174, a typical two-rowed barley variety with long thin and sterile lateral spikelet, and also long awn, to obtain the F_1_ seeds. It was found that spikes of the F_1_ plants showed intermedium type, awns of which were long and same as wild-type plants (MM), but were tighter than wild-type awns. Lateral spikelets of F_1_ plants were fatter than wild type and crooked, but bilateral empty glumes of lateral spikelets were the same as *rls* (mm) (Fig. 1b–e). Individuals with the same phenotype as F_1_ plants were recorded as “mM” in this study (Fig. 1b–e). While the F_2_ population showed a segregation of the MM, mM and mm type spikes, which included 268, 462 and 286 plants, respectively. Chi-test showed that the number of MM, mM and mm type plants in the F_2_ population fitted a ratio of 1:2:1. Moreover, all the F_2:3_ families derived from MM type F_2_ individuals had homozygous wide type spikes, all the F_2:3_ families derived from mM type F_2_ individuals showed a segregation of three types spikes, while all the F_2:3_ families derived from mm type F_2_ individuals had homozygous mutant type spikes, which confirmed that this changed spike morphology in *rls* was controlled by a single semi-dominant gene, and was designed as *HvRLS* in this study.

### Preliminary mapping of *HvRLS* candidate gene via BSA based on RNA-Seq

**Fig. 3:**
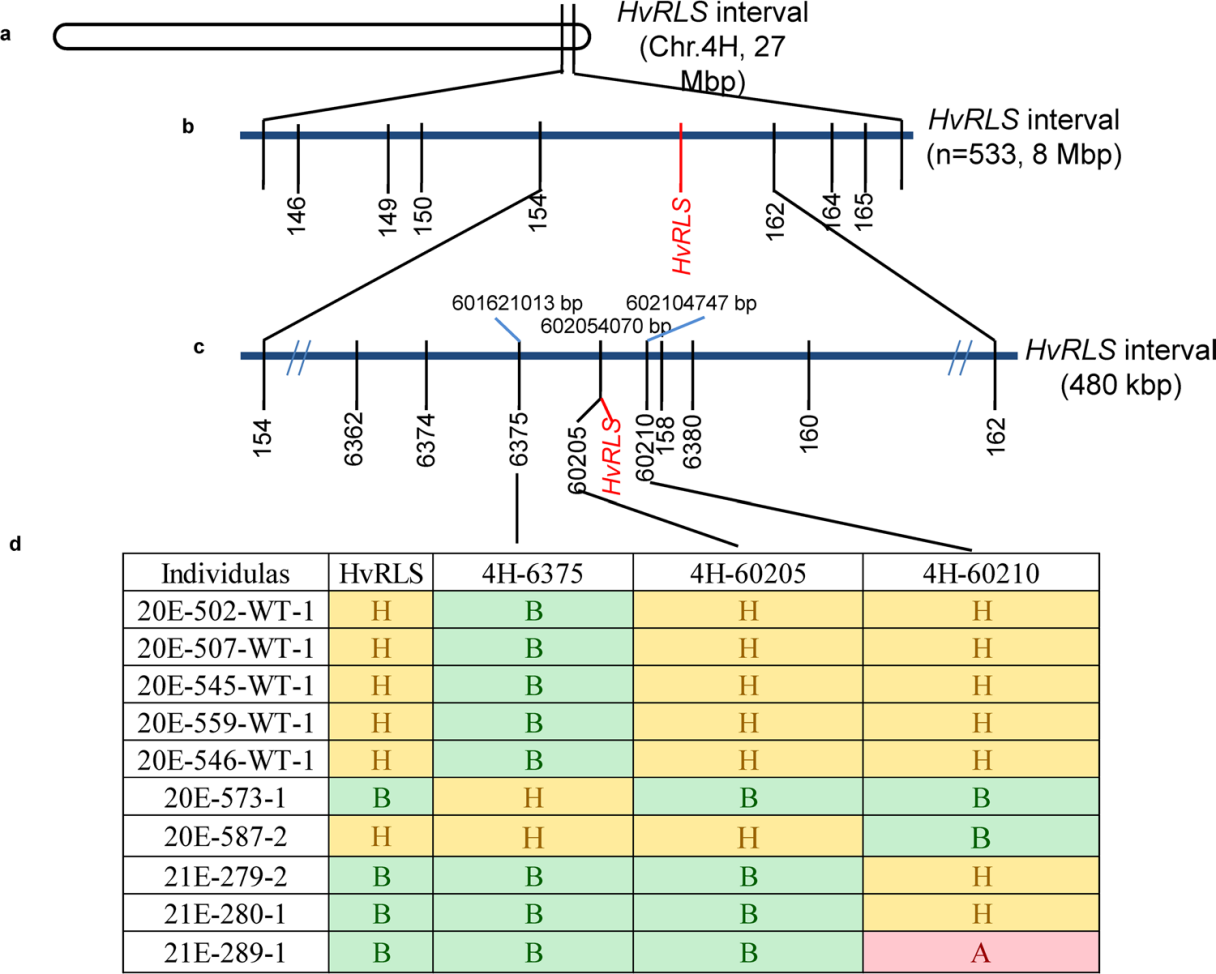
Map-based cloning of *HvRLS.* **a** Initial mapping; **b**, **c** fine mapping; **d** phenotype and genotype of recombinant lines. Note: A, B and H in Fig. 3d stand for homologous wild-type allele MM, homologous mutant allele mm and heterozygous allele mM, respectively

To map the causal gene for *rls* phenotype, BSA based on RNA-Seq was initially conducted using mutant pool and wild-type pool derived from 60 heterozygous F_2:3_ families (progenies of mM type F_2_ individuals). Totally 494 SNPs/Indels with *p* < 1e−10 and AFD > 0.6 were identified as potential linkage with the phenotype, half (246) of which could be anchored to the end of long arm of chromosome 4H (Totally 647.06Mbps), falling into the interval of 619Mbp-646Mbp (MorexV1 assembly) (Fig. 3a). However, only 23, 40, 67, 42, 32 and 40 SNPs/Indels were on chromosome 1H, 2H, 3H, 5H, 6H and 7H, respectively, indicating that *HvRLS* gene was on chromosome 4H.

To validate the BSR analysis and fine map the gene, totally 15 polymorphic markers (Supplementary Table S1) in the target region were developed according to the BSR-seq results. These markers were then used to screen the genotypes of *rls* and Yan 03174 as well as the F_3_ and F_4_ plants derived from mM type F_2_ and F_3_ individuals, respectively. Based on PARMS, totally 505 mm, 16 mM and 12 MM type plants from F_3_ and F_4_ populations were genotyped and analyzed. Finally, *HvRLS* gene was mapped between 4H-6375 and 4H-60210 (Fig. 3b, c), which was located at 601,621,013 bp and 602,104,747 bp on chromosome 4H according to MorexV3 assembly (Mascher et al. 2021), spanning about 480 kb. Out of the 533 F_3_ and F_4_ individuals, six and four recombinant events were identified for 4H-6375 and 4H-60210, respectively (Fig. 3d). The most interesting was that the genotypic data of 4H-60205, representing a single nucleotide polymorphism (SNP) G/A, co-segregated with MM, mM and mm phenotype in the segregate populations (Fig. 3d). The co-segregation was also confirmed in the F_2_ population derived from *rls* and Yan 03174, including 268 MM, 462 mM and 286 mm individuals.

### Cloning of *rls* candidate gene

**Fig. 4:**
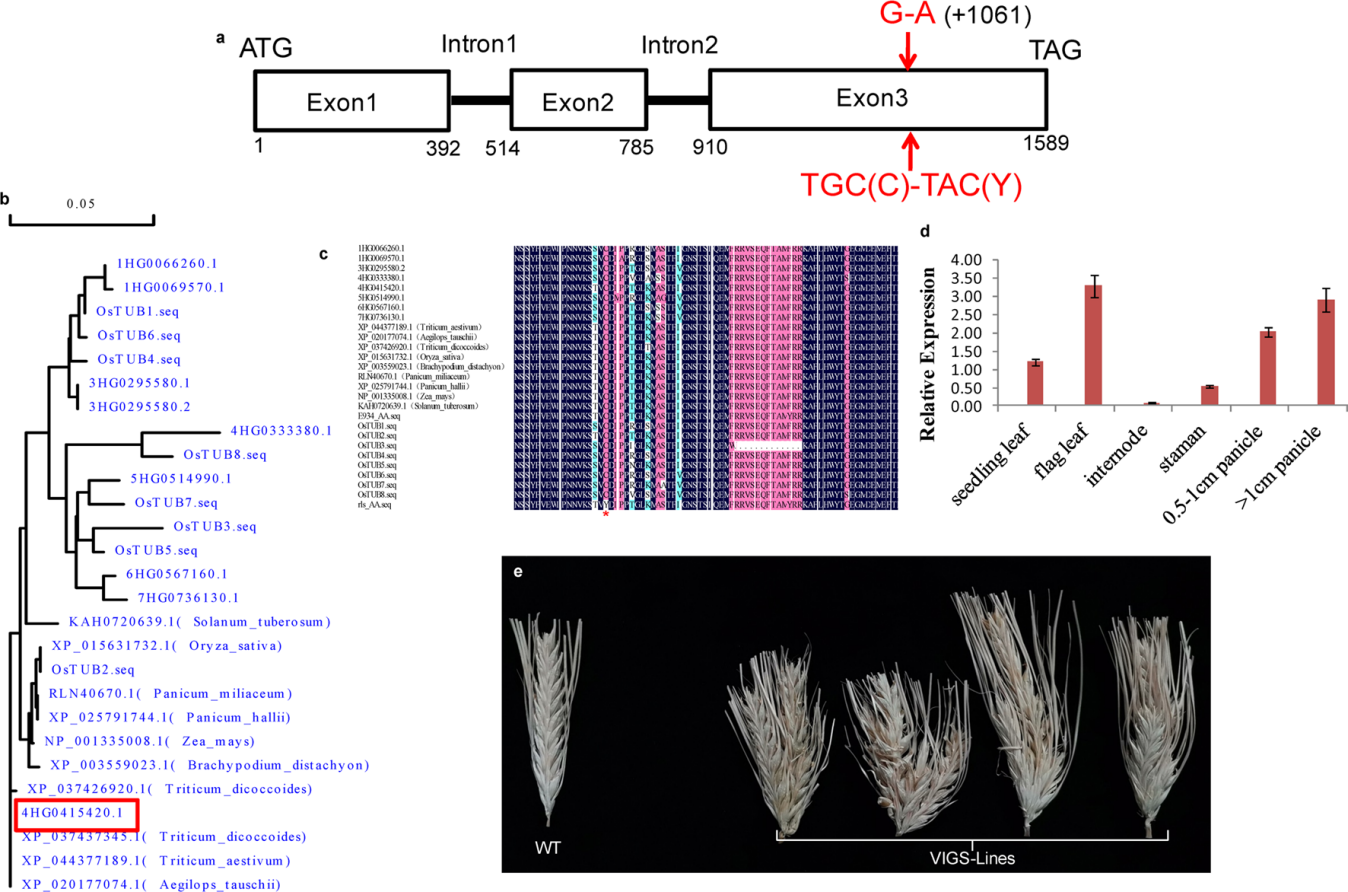
Sequence, expression and functional analysis of *HvTUB8*. **a** Gene structure of *HvTUB8* (Red indicates the G-A and C-Y substitution on the third exon); **b** phylogenetic analysis of HvTUB8 and its homologue genes; **c** partial alignment of HvTUB8 and its homologue genes (red star indicates the C-Y substitution); **d** spatio-temporal expression of *HvTUB8* in E934; **e** spikes of BSMV:*HvTUB8*-infected and non-infected E934 (WT) (color figure online)

Further analysis showed that there were 17 high-confident genes (Table 1) in this 480 kb target region. It was very interesting that the G to A substitution represented by 4H-60205 was within a *β-TUBULIN* gene. Further BLAST analysis showed that this *β-TUBULIN* gene was the previously annotated *HvTUB8.* According to the updated barley genome, genomic sequence and coding region of *HvTUB8* was 1589 bps and 1344 bps in length, respectively, which had three exons and encoded a polypeptide composed of 447 amino acid residues harboring the conserved “TUBULIN beta chain” domain (1–430/447), and the G-A variation was on the third exon (+ 1061) of *HvTUB8* (Fig. 4a).

**Table 1:** High-confident genes in the target region based on MorexV3 assembly

Gene ID	Location on chromosome 4H (Mbps)		Gene description
	Start	End	
4HG0415300	601627654	601629075	RNA-directed DNA polymerase (reverse transcriptase)-related family
4HG0415310	601641518	601641826	Homer protein homolog
4HG0415320	601652557	601652931	Non-structural protein NS1
4HG0415330	601729791	601733093	Cytochrome P450 family cinnamate 4-hydroxylase
4HG0415340.1	601760190	601765625	ATP-binding cassette transporter subfamily A
4HG0415350	601810049	601810648	DNA topoisomerase
4HG0415360	601811485	601812995	F-box family protein
4HG0415370	601821300	601821821	Villin-like 1
4HG0415380	601823536	601823943	Leucyl/phenylalanyl-tRNA–protein transferase
4HG0415390	601825582	601825959	Protein TolB
4HG0415400	601853564	601854058	Myosin heavy chain, embryonic smooth protein
4HG0415410	602046060	602048127	AT hook motif DNA-binding family
4HG0415420	602053484	602055456	Tubulin beta
4HG0415430	602085760	602087165	MYB-related transcription
4HG0415440	602095267	602095641	RNA-directed DNA polymerase (reverse transcriptase)-related family
4HG0415450	602102008	602102721	Receptor-like kinase
4HG0415460	602104336	602106756	Receptor-like protein kinase

To validate the co-segregated G-A substitution, full length coding regions of *HvTUB8* were cloned from genomic DNA and cDNA of young panicles of E934 and *rls* subsequently. Both of the sequences from gDNA and cDNA confirmed the G to A mutation in *rls*, while no other variation was detected in coding region between E934 and *rls*. Moreover, there was no difference between the sequence of 1000bps upstream region from E934 and *rls* either*,* whereas coding sequences and their expression in immature spikes of the other 16 genes showed no difference between E934 and *rls*. Further study exhibited that the cysteine (C) at amino acid position 354 of HvTUB8 was replaced by a tyrosine (Y) in *rls* due to the G-A substitution (Fig. 4a).

### Phylogenetic analysis of β-TUBULIN proteins in plant

BLASTP and BLASTN of the wide-type HvTUB8 protein was then conducted online, and homologue proteins from barley and its relatives, such as *Triticum dicoccoides*, *Triticum aestivum*, *Aegilops tauschii*, *Oryza sativa* and *Zea mays*, were downloaded. As shown in Fig. 4b, these proteins were highly conserved among different species, which showed 95% to 100% similarity to each other. Phylogenetic analysis indicated that HvTUB8 belonged to a mini cluster that only included members from *Triticum* and its ancestor (Fig. 4b). HvTUB8 protein showed 100% and 96.89% similarity to its counterparts from *Triticum dicoccoides* (XP_037437345.1) and *Oryza sativa* (OsTUB2), respectively. Though the Cys to Try substitution mentioned above located in a region that was not quite conserved as compared with other regions, the wide type Cys was highly conserved among barley and its relatives, the substitution could only be detected in *rls* (Fig. 4c).

### Spatial and temporal expression of *HvTUB8* in E934

Then spatial and temporal expression profile of *HvTUB8* was analyzed in 10 tissues of E934 at different developmental stages. Result showed that expression pattern of *HvTUB8* was highly tissue-specific. It mainly expressed in young panicles and leave, and a relative lower expression was also detected in stamen and internode below the spike at the heading stage (Fig. 4d). However, it was hardly detected in other tissues.

### Haplotype analysis of *HvTUB8* gene in barley accessions

The co-segregated maker QDD4H-60205, which represented the G to A mutation in *HvTUB8,* was also applied to screen a panel of 400 barley accessions collected from different countries with typical two- or six-rowed type spikes. As expected, all of these accessions had the “G” type allele as E934. To further explore the natural diversity of *HvTUB8*, genetic variation of the gene was analyzed in the 20 barley accessions together with E934 using the barley pan-genome data. Coding region from the start codon to stop codon was extracted from 19 accessions except for “Igri”, with nine bps deletion at the very beginning of the gene. It was also found that all these 20 accessions had the same allele (G) as E934 at + 1061. Additionally, a total of 14 other SNPs were detected in coding region of *HvTUB8*, which resulted in seven haplotypes (Supplementary Table S2) in the 20 accessions, while *HvTUB8* in E934 was distinctive and different from all the seven haplotypes. In addition to coding region, 1000 bps upstream regions of *HvTUB8* were analyzed between these 19 accessions except for HOR3365. Abundant variations were also detected in 1000 bps upstream of this gene. However, none of these haplotypes was associated with barley row type.

### Functional characterization of *HvTUB8* by BSMV-VIGS

To confirm the participation of *HvTUB8* in barley spike development, we used BSMV-VIGS to silence the gene in wild-type E934 plants. Control infections with BSMV indicated that viral infection did not affect spike morphology of barley, and with BSMV-*TaPDS* indicated infection and gene-silencing efficiency, as shown by bleached lines on infected leaves. Expression analysis of the target gene in leaves of these BSMV:*HvTUB8*-infected plants suggested that expression of *HvTUB8* was reduced about 70% as compared with in non-transformed E934. As shown in Fig. 4e, irregular spikes were consistently observed in the four survived plants infected with BSMV:*HvTUB8*, and extra spikes were produced along the main spike, which was different from the known barley branched spikes. However, no other difference, like grain shape, awn length, leaf color was observed in BSMV:*HvTUB8*-infected E934 plants as compared with the non-treated E934. Spikes of the plants infected by BSMV control showed no difference with the wide type. The results suggested that the wild-type *HvTUB8* gene is essential for maintaining the integrity of barley spike.

### Subcellular localization of HvTUB8 and Hvtub8 proteins

**Fig. 5:**
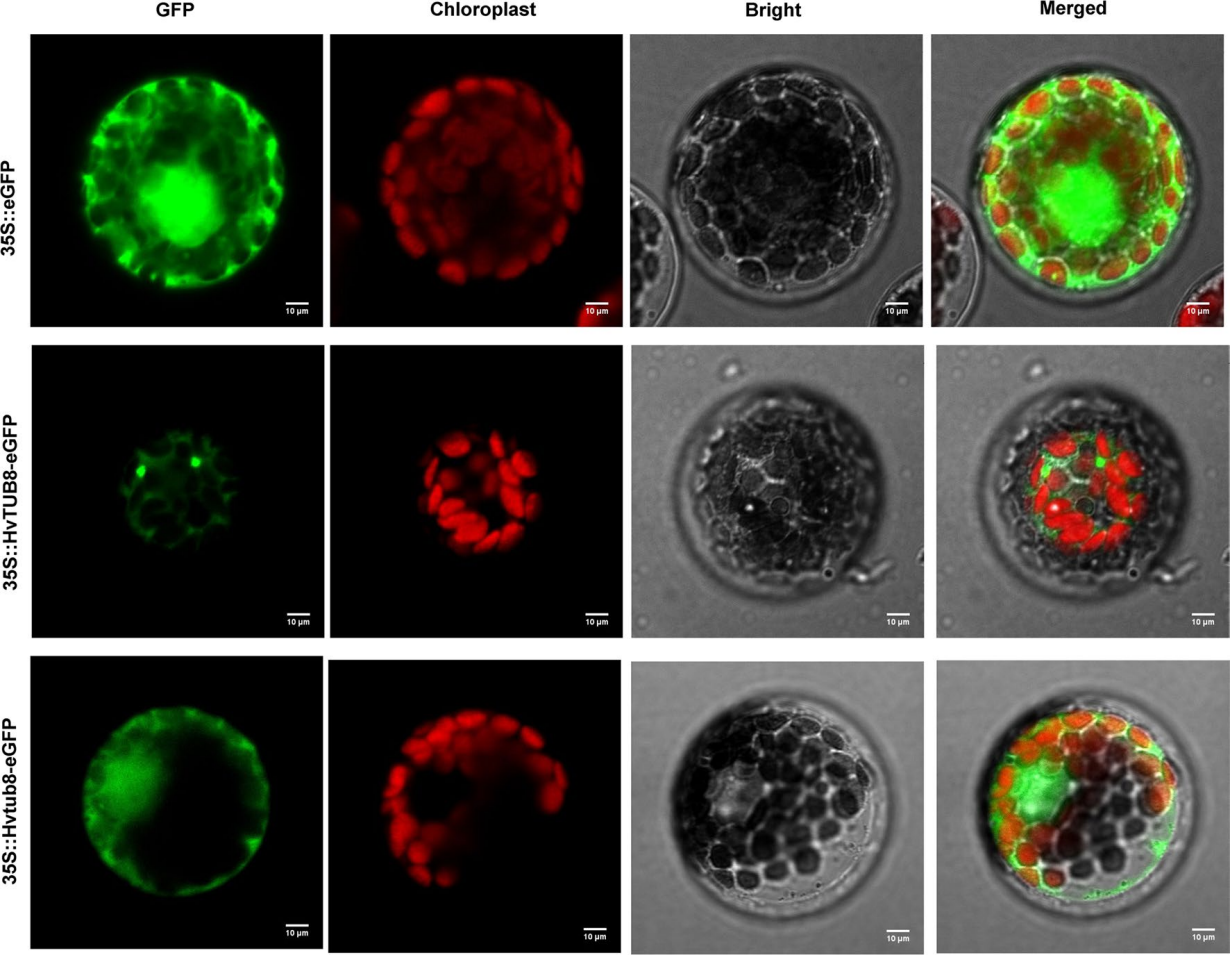
Subcellular localization of HvTUB8 protein. 35S::eGFP indicates the expression of GFP protein in wheat protoplasts as the negative control. 35S::HvTUB8-eGFP indicates the cytoplasm localization of wild-type HvTUB8 protein in wheat protoplasts. Green is GFP signal. Bright is the bright light. Merged indicates the confused with the green signal and the bright light. Bars: 10 μm

To demonstrate the subcellular localization of HvTUB8 protein, both the full-length *HvTUB8* and the mutated *Hvtub8* were fused with GFP reporter and expressed in wheat protoplasts. As shown in Fig. 5, in the positive control cells expressing 35S::GFP protein only, GFP signal was observed throughout the cells, but the single amino acid change of HvTUB8 caused change of subcellular localization of the protein in wheat protoplasts. In the cells expressing 35S::HvTUB8:GFP protein (Fig. 5e–h), a weak signal was detected in cytoplasm, and a strong signal was detected in a few of granular organelles. However, in the cells expressing the Hvtub8 fused GFP protein, a strong green florescence signal was detected in both the nucleus and cell membrane.

### Transcriptome profiling reveals the downstream genes regulated by *HvRLS*

To interrogate the downstream signaling pathways regulated by *HvRLS* and their contributions to barley development, RNA-Seq was performed on 0.5–1 cm young panicles of *rls* and E934 with three biological replicates. Totally, 40,892 expressed genes were detected with 441 genes being differentially expressed (DEG) in *rls* relative to E934, but none of the 17 genes (including *HvTUB8*) within the mapping interval showed DEG between E934 and *rls*, and more than three quarters (340) of these differentially expressed genes (DEGs) were up-regulated by the mutation of *HvRLS* (Supplementary Table S3).

Then, expressions of known barley lateral spikelet development regulating genes, including *VRS1*, *VRS2*, *VRS3*, *VRS4* and *VRS5,* were further analyzed, but none of them were altered in *rls* as compared with in wild type*.* Two LOB domain-containing protein genes on 3H (HORVU.MOREX.r3.3HG0233680) and 4H (HORVU.MOREX.r3.4HG0414380) were up-regulated by threefold and 7.6 fold, respectively, and a Homeobox-leucine zipper family protein (HORVU.MOREX.r3.2HG0189890) was also up-regulated by four folds in *rls*. However, the two LOB domain-containing genes only showed 30.35% and 33.16% similarity to *VRS4*/*HvRA2* in the coding region, while the Homeobox-leucine zipper family protein showed low level (40.55%) of similarity to *VRS1*, respectively.

**Fig. 6:**
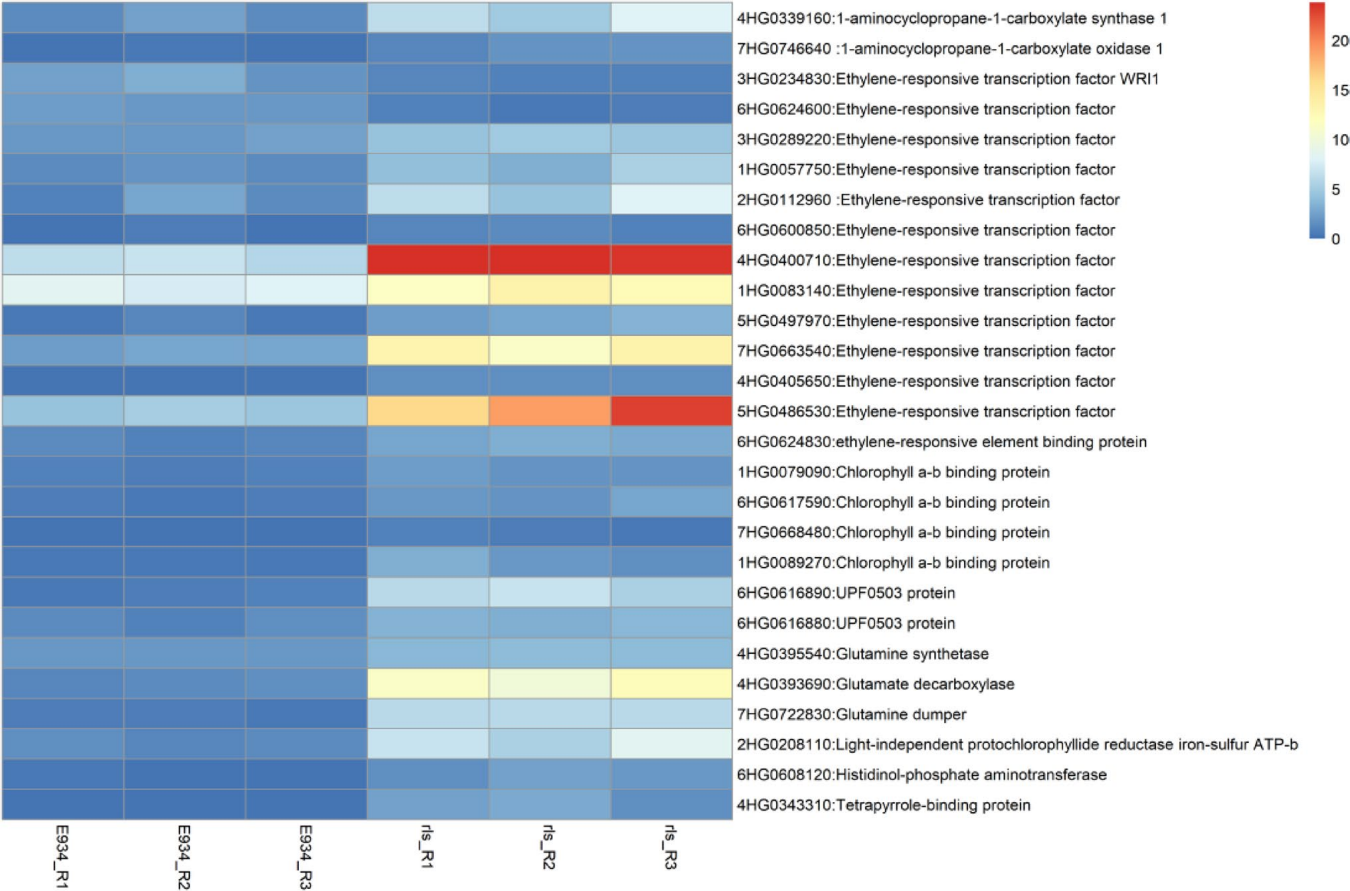
Expression of DEGs in ethylene and chloroplast pathways

Further GO enrichment analysis of the 441 DEGs showed that about half of the DEGs (213) could be anchored to known GO terms (Supplementary Table S4). Totally, 34, 5 and 1 GO terms were enriched for Biological Process, Molecular Function and Cellular Component, respectively. It was found that phytohormones homeostasis was extensively disturbed in *rls*. Notably, a 1-aminocyclopropane-1-carboxylate oxidase gene and a 1-aminocyclopropane-1-carboxylate synthase gene in “ethylene biosynthetic process”, together with 12 of the 14 ethylene-responsive transcription factors in “ethylene-activated signaling pathway” were significantly up-regulated in *rls* (Fig. 6). Additionally, genes responsive to other phytohormones were also enriched in DEGs, including salicylic acid, brassinosteroid and gibberellic acid, and most of them were transcription factors, such as WRKY and zinc finger proteins. Moreover, the RNA-Seq data also witnessed a large amount of other transcription factors being regulated by mutation of *HvTUB8*, such as NAC, F-box, MYB, CRT-binding factors, bHLH, MADS-box and so on. The majority of them were up-regulated in *rls*. On the other hand, expressions of 32 genes encoding various protein kinases (LRR, WAK etc.) were changed in *rls*, with 27 and 5 being up-regulated and down-regulated, respectively.

Considering the difference of leaf color and ultrastructure of chloroplast of E934 and *rls*, chlorophyll biosynthesis and metabolism-related genes were analyzed subsequently. It was found that expressions of 11 genes participating in chloroplast synthesis and metabolism were changed by the mutation of *HvRLS* in *rls,* nine of which were also up-regulated (Fig. 6).

## Discussion

### *rls* is a rare and distinctive resource for barley study

Functional characterization of genes and genetic mechanisms involved in barley inflorescence performance has been well elucidated since the identification of *VRS1*, which was a negative regulator of barley lateral spikelet development. At least five *VRSs* (*VRS1-5*) have been identified since then. Variation at *VRS1* was sufficient to control the formation of two-rowed or six-rowed barley (Ramsay et al. 2011b), while induced loss function of alleles of any of the other genes could cause complete to intermediate gains of lateral spikelet fertility (Zwirek et al. 2019). Recently, *HvAP2L-H5* was identified to be required for barley inflorescence indeterminacy and spikelet determinacy (Zhong et al. 2021)*.* Except for the *vrs* mutants mentioned above with partial or completed sterility of the lateral spikelet, several *intermedium*-spike barley mutants were also identified with significant different lateral spikelet morphology and size (Franckowiak and Lundqvist 2012; Youssef et al. 2020). For example, spike of *int-f* appeared six-rowed, but the lateral spikelets were small and pointed (Gustafsson and Lundqvist 1980). Most of these barley row-type-related genes have pleiotropic effects on shoot branching, grain number per spike and thousand grain weight, either background dependent or independent (Liller et al. 2015). *rls* in this study was mainly characterized as a mutant in spike morphology, grain shape, plant height and leaf color. Particularly, lateral spikelets of *rls* were completely sterile but much shorter and fatter than wild-type ones, which were different from those known *vrs* mutants and were described as “round” in the present study. Though the pointed lateral spikelets were also observed in some *intermedium* mutants, *rls* has pleiotropic variations of spike, grain, stem, leaf and awn, suggesting that the underling gene was different in controlling barley development. Therefore, identification and functional characterization of the causal gene will be important to fully understand the complex network in barley spike, grain and leaf development, and thus ultimately provide targets for understanding and exploiting natural or induced genetic diversity toward improving barley yield potential and grain physical quality.

### HvTUB8 as a candidate gene for rls phenotype

Based on BSR and fine mapping, *HvRLS* was anchored within a 480 kb interval with 17 annotated genes on chromosome 4H. The gene was co-segregated with a marker 4H-60205 (Fig. 3d). Further analysis found that a G/A substitution for the marker 4H-60205 was located at the third exon of *HvTUB8*, a member of *β-TUBULIN* gene family. We further tested the G/A substitution in a natural population with 400 accessions and analyzed the gene variations in the barley pan genome panel (Jayakodi et al. 2020). The “A” allele of *HvTUB8* could not be found in the natural barley population and is specific to *rls*, indicating that it is an artificial allele induced by chemical and physical mutation. Transcriptomic sequencing analysis further confirmed that the G/A substitution was the only variation in the coding sequences of the 17 genes within the interval between the parental line E934 and the mutant *rls*. As there was no detectable gene expression difference or alternative splicing between E934 and *rls*, any potential sequence variations in the non-coding regions within the interval are unlikely to play an important role for the observed phenotype in *rls*. These results indicated *HvTUB8* as the candidate gene and the G/A substitution as the casual mutation, which lead to Cys354Tyr substitution in *rls*.

Plant TUBULIN is a dimeric protein that contributes to formation of microtubules, and it is highly conserved and composed primarily of monomeric globular polypeptides designated as *α-* and *β-TUBULINs* (Breviario et al. 2013). Microtubules are a conserved cytoplasmic structure found in all eukaryotes. They function in essential roles ranging from cellular morphogenesis to cell movement, cell division, polarity of growth, cell-wall deposition, intracellular trafficking and communications (Breviario et al. 2013; Koo et al. 2009). Cysteine residues often play essential roles in protein structure and function by conferring stability through disulfide bond formation, maintaining proper maturation and localization through protein–protein intermolecular interactions, or providing a thiol group for reactions with molecular substrates (Meitzler et al. 2013). In potato, there were 12 and 11 cysteines in α- and β-TUBULINs, respectively. These cysteine residues play vital role in accessibility of tubulin for modification by benomyl, colchicine and GTP binding (Koo et al. 2009). There were 12 cysteines in the wide type HvTUB8 protein. HvTUB8 homologue proteins from other species showed high level of similarity. Previous studies showed that the Cysteine 354 of β-TUBULIN was essential for binding of colchicine (Bai et al. 1996, 2000) and acrolein (Uemura et al. 2019), and mutation of this cysteine in yeast β-TUBULIN could result in cold-stable microtubules (Gupta et al. 2010). Our result demonstrated that the Cys354Tyr substitution resulted in changes of subcellular location of the protein. The mutant Hvtub8 protein was diffused to nuclear and membrane, while the wild-type HvTUB8 was concentrated in cytoplasm and a few of granular organelles (Fig. 5). The results indicated that mutation of the Cys354Tyr changed the protein location and interaction between HvTUB8 and its counter partner subsequently. This result is consistent with the rice *paa3* protein which encoded a H+ -ATPase and controlled panicle development and seed size in rice (Yang et al. 2021). The Arg258Gln substitution caused by a G-A mutation in rice receptor-like kinase *CR4* could also disturb its subcellular localization, and lead to the change of grain size and shape in *mis2* mutant (Yan et al. 2020). In wheat, the grain weight controlling gene *GW7* co-localized with α- and β-TUBULIN proteins in the cells, which was involved in the pathways regulating cell division and organ growth (Wang et al. 2019b). We speculated that the Cys354Tyr substitution in *rls* altered formation of tubulin complex and microtubules and resulted in changes of cell morphology and arrangement in spikelet hull and leaf, and thus provided further evidence to support *HvTUB8* as the functional gene.

In plants, cortical microtubule arrays play a critical role in plant cell shape determination by defining the direction of cell expansion. Thus, TUBULIN genes are critical in all kinds of physiological and developmental processes, including grain shape. For example, three rice α-TUBULIN genes *TubA1*, *TubA2* and *TubA3* showed significantly different expression in flowers, roots and coleoptile segments treated with auxin (Qin et al. 1997), while members of *β-TUBULIN* gene may be responsible for chilling (Jeong et al. 2021) and salt tolerance (Jin et al. 2021) of rice. Rice auxin-inducible and microtubule-localized protein OsIQD14 could affect grain shape by regulating microtubule rearrangements in rice hull cells (Yang et al. 2020). What is more, members of *α-TUBULIN* were not only the causal gene for *Small and round seed 5* (*Srs5*) (Segami et al. 2012), but also for *Twisted dwarf 1* (*Tid1*) (Sunohara et al. 2009). These results are consistent with our observation that *rls* mutant has round and short grain and thus support *HvTUB8* as the functional gene.

To further confirm *HvTUB8* as the functional gene, we performed virus-induced gene (VIG)-silencing experiment. Silencing of *HvTUB8* resulted in alteration of barley spike morphology (Fig. 4e), which confirmed that *HvTUB8* was essential for maintaining barley spike integrity. However, the resulting spike phenotype was not exactly same as the *rls* mutant. This could be due to the following two facts: (1) the gene-silencing inoculation was conducted at the two-leaf stage, such early gene silence was effective to interfere the inflorescence development, but may not impact for the grain development at later stage; thus, we could only see abnormal spike but not the round grain; (2) VIG only partially inhibited the gene expression, but the *rls* resulted in cellular location change of the protein due to loss of the Cys; thus, the VIG result was in-consistent with the mutant. More research is required to confirm *HvTUB8* as the functional gene through precision gene editing, e.g., prime-editing.

### *HvRLS *participates in barley lateral spikelet development independent of known *VRS* gene

Being consistent with the hypothesis of gain-of-function of *HvRLS*, the majority (77%) of DEGs between E934 and *rls* were up-regulated in *rls.* Among those known VRS genes, *VRS1* acts as a downstream integrator of *VRS3*, *VRS4* and *VRS5* to repress lateral spikelet fertility and has been considered as the switch between two-rowed and six-rowed, while *VRS3* functions as an epigenetic modifier of row-type gene function (Zwirek et al. 2019). However, none of these reported lateral spikelets governing genes was differentially expressed in *rls* mutant, suggesting that *HvRLS* regulates spike development in a *VRS*-independent manner. As a novel gene in shaping barley spike morphology, mutation of *HvRLS* likely induced the expression of two LOB domain-containing protein genes and a Homeobox-leucine zipper family protein gene, which show low level sequence similarities with *VRS4*/*HvRA2* and *VRS1*, respectively. Moreover, three *MADS-box* genes but not the *HvMADS56* were also identified to be differentially expressed between E934 and *rls*, two of which were also up-regulated in the mutant. These results suggested that *HvRLS* regulate barley spike development through a different pathway but share similar regulating mechanisms with other known lateral spikelet determining genes.

### *HvRLS* participates in barley development via ethylene signaling and chlorophyll biosynthesis

Distribution and concentration of different hormones play vital roles in barley inflorescence morphology. Loss of function of *VRS2* is associated with auxin and cytokinin imbalances along the spike (Youssef et al. 2016). As one of the most important phytohormones, ethylene plays a critical role not only throughout the plant life cycle (Larsen 2015), but also in their response to biotic and abiotic threatens (Dubois et al. 2018). *Arabidopsis thaliana* mutants in ethylene signaling pathway presents notable alterations in seed shape (Robert et al. 2008). It has recently been proved that ethylene biosynthesis also plays a key role in maize ear elongation and kernel yield recently (Ning et al. 2021). Rice U-box E3 ubiquitin ligase gene positively regulates seed cell division and elongation through multiple hormone pathways, including ethylene (Wang et al. 2017a, 2017b). In this study, some key genes involving in ethylene biosynthesis and signaling, such as ACC synthase, ACC oxidase, ethylene-responsive transcription factors, were distinctively up-regulated upon the mutation of *HvRLS* in *rls*, suggesting potential regulatory roles of these genes in barley spike development. Actually, it has been proved previously that ethylene signaling pathways can initiated transfer cells morphology and control cell shape and vesicle transport of barley (Thiel et al. 2012).

Except for spike morphology, leaf color of *rls* was darker than that in E934. Accordingly, number and size of chloroplasts were increased in *rls*, which was also consistent with the higher chlorophyll concentration detected in *rls* seedling leaves when compared with that in E934. A previous study has confirmed that functional attenuations in chlorophyll biosynthesis-related genes, including *HrHEMA*, *HrPOR* and *HrCAO*, induce leaf color and chloroplast structure changes in *Hosta* (Zhang et al. 2021). Though transcriptomic analysis was conducted on young panicles of *rls* and E934 in this study, genes being responsible for chlorophyll biosynthesis and metabolism were indeed up-regulated in *rls*, such as Glutamine synthetase and decarboxylase genes, chlorophyll a-b binding protein coding genes, aminotransferase genes, protochlorophyllide reductase genes. This indicated that *HvRLS* may control leaf color and chloroplast structure through chlorophyll biosynthesis and metabolisms.

## Supplementary Information

Below is the link to the electronic supplementary material.

Supplementary file1 (XLSX 86 KB)

## Data Availability

All data supporting the findings of this study are available within the paper and within its supplementary materials published online.
